# EFFECTIVENESS OF A SIMULATION-BASED EMPATHY ENHANCEMENT PROGRAM WITH MINDFULNESS ON SOCIAL WORKERS

**DOI:** 10.1093/geroni/igz038.1093

**Published:** 2019-11-08

**Authors:** Areum Han, Taehee Kim

**Affiliations:** 1 Yonsei University, Wonju, Gangwon, Korea, Republic of; 2 Yonsei University Wonju Severance Christian Hospital, Wonju, Gangwon, Korea, Republic of

## Abstract

Empathy enhancement programs and mindfulness-based practices may reduce care practitioners’ burnout and stress while increasing satisfaction, caring efficacy, and well-being. No study has been conducted to measure the effectiveness of a simulation-based empathy enhancement program combined with mindfulness practice on professionals working with older adults living alone. This study, therefore, assessed the effectiveness of a simulation-based empathy enhancement program with a brief mindfulness practice session on social workers working with older adults living alone. This study was a quasi-experimental study involving 105 social workers in South Korea. The experimental group received a simulation-based empathy enhancement program with mindfulness practice, and the attention control group watched a 30-minute-long educational video about empathy. Data were collected prior to the intervention and at two weeks after the intervention using self-reported questionnaires measuring empathy, caring efficacy, psychosocial stress, compassion satisfaction, burnout, and secondary traumatic stress. The experimental group had significantly lower levels of psychosocial stress compared to the attention control group. Both groups showed significant improvements in empathy but in different empathy measures. Also, the experimental group only showed significantly lower levels of burnout and secondary traumatic stress after the intervention while the attention control group only showed significant improvements in compassion satisfaction and caring efficacy. Although between-group differences were found in psychosocial stress only, pre-and post-test differences in different outcome measures from experimental and attention control groups indicate limited but possible effectiveness of each of the empathy enhancement programs on people in caring professionals.

